# Micro/Nano Structural Tantalum Coating for Enhanced Osteogenic Differentiation of Human Bone Marrow Stem Cells

**DOI:** 10.3390/ma11040546

**Published:** 2018-04-03

**Authors:** Ding Ding, Youtao Xie, Kai Li, Liping Huang, Xuebin Zheng

**Affiliations:** 1 Key Laboratory of Inorganic Coating Materials CAS, Shanghai Institute of Ceramics, Chinese Academy of Sciences, 1295 Dingxi Road, Shanghai 200050, China; dingding@student.sic.ac.cn (D.D.); xieyoutao@mail.sic.ac.cn (Y.X.); likai@mail.sic.ac.cn (K.L.); lipinghuang@mail.sic.ac.cn (L.H.); 2 University of Chinese Academy of Sciences, 19 Yuquan Road, Beijing 100049, China

**Keywords:** micro/nano structure, tantalum coating, osteogenic differentiation, nanotopographical modification, plasma spraying

## Abstract

Recently, tantalum has been attracting much attention for its anticorrosion resistance and biocompatibility, and it has been widely used in surface modification for implant applications. To improve its osteogenic differentiation of human bone marrow stem cells (hBMSCs), a micro/nano structure has been fabricated on the tantalum coating surface through the combination of anodic oxidation and plasma spraying method. The morphology, composition, and microstructure of the modified coating were comprehensively studied by employing scanning electron microscopy (SEM), X-ray diffraction (XRD) as well as transmission electron microscopy (TEM). The effects of hierarchical structures as well as micro-porous structure of tantalum coating on the behavior for human bone marrow stem cells (hBMSCs) were evaluated and compared at both cellular and molecular levels in vitro. The experimental results show that a hierarchical micro/nano structure with Ta_2_O_5_ nanotubes spread onto a micro-scale tantalum coating has been fabricated successfully, which is confirmed to promote cell adhesion and spreading. Besides, the hierarchical micro/nano tantalum coating can provide 1.5~2.1 times improvement in gene expression, compared with the micro-porous tantalum coating. It demonstrates that it can effectively enhance the proliferation and differentiation of hBMSCs in vitro.

## 1. Introduction

Metallic implants such as titanium and its alloys, which possess excellent mechanical properties and biocompatibility, have been extensively used to treat hard tissue disorders [1,2]. However, titanium and its alloys are relatively bio-inert which may hardly induce rapid osteogenesis and firm fixation [3,4]. To improve the rapid osseointegration to bone tissue, surface modification may be one of the important methods. Many studies have revealed that the surface characteristics of implant such as surface roughness and topography can effectively regulate the biological performance of implants [5,6].

Tantalum (Ta) coating as an effective surface modification method on the implants has attracted great attention in the past several decades due to its excellent biocompatibility [7,8,9,10,11]. Ta coating with micro-porous structures fabricated by plasma spraying has been demonstrated that it can promote in-growth of bone tissue as well as formation of mechanical interaction with host bone tissue [12,13,14,15,16]. Nevertheless, these kinds of micro-rough surfaces can depress the early cell responses and a smaller bone mass accumulated compared with a smooth surface [17,18,19,20,21,22,23]. Recently, nano structuring modification has been used to induce fast osteointegration [24]. Nano-porous surface can effectively regulate the growth and orientation of cells from the biomechanical perspective, which is beneficial for the cytoskeleton alignment and rearrangement [25,26]. Moreover, from the physical perspective, nano-porous structure closely resembles the natural extracellular matrix [26,27,28]. Wang et al. have proved that the increased growth, vitality of osteoblast and mesenchymal stem cells on Ta_2_O_5_ nanotubes surface compared to the flat tantalum [29]. It is envisioned that these Ta_2_O_5_ nanotube arrays can imitate complicated geometries of the human tissue, providing a porous network for development and maintenance of cells [30].

Current trends in the implant morphology modification field highlight the hierarchical micro/nano structure to maintain effective and rapid osseointegration [31,32]. Compared with nano-porous structure, hierarchical micro/nano structure can promote cell functions [33,34]. Besides, designing a hierarchical micro/nano structured surface similar to natural bone tissues has great potential to improve the cell functions, from a biomimetic viewpoint [35]. Cell adhesion could be enhanced through micro-scale topography while cell proliferation could be promoted through nanotopographic surfaces. This effect in human marrow stem cells (hMSCs) have been demonstrated by Zheng et al. using micro/nano Ti structure [23,36]. In addition, titanium oxides formed in situ via anodization technique have many advantages such as high surface-to-volume ratio and controlled dimensions, which has also been demonstrated the beneficial effects to cell response [37,38].

Numerous studies have reported the fabrication methods and effect of titanium surfaces with hierarchical micro/nano-porous structure. However, few studies have been devoted to the construction and modification with micro/nano structure of tantalum coating to enhance its osteogenic differentiation of human bone marrow stem cells (hBMSCs). In our previous studies, the plasma-sprayed Ta coating with micro-scale displayed improved cytocompatibility and osteogenic differentiation properties compared with Ti coatings. To further enhance the osteogenic performance of the tantalum coating, we herein propose a novel way of combining the plasma spraying and anodization technique to develop a micro/nano tantalum (MNT) with hierarchical structure. Tantalum oxides have been demonstrated that formed in situ via anodization technique. The surface of micro-porous tantalum (MT) coating fabricated by plasma-sprayed method was used as control group for protein adsorption and cell culture experiments in vitro.

## 2. Materials and Methods

### 2.1. Preparation of Plasma-Sprayed Tantalum Coating

Pure tantalum (particle size of about 70 μm) powders with a purity of 99.99% commercially available were used in the present work. The tantalum coating with thickness of 300 μm was fabricated on pure titanium plates with dimensions of Φ10 mm × 2 mm by vacuum plasma spraying (VPS) system (Sulzer Metco, Wohlen, Switzerland).

### 2.2. Fabrication of Ta_2_O_5_ NTs by Anodization

The as-deposited tantalum coating (Φ10 mm × 300 μm) on Ti plates was firstly ultrasonicated in acetone and ethanol. Then, the coating was connected to the anode while a platinum foil (10 mm × 10 mm × 0.5 mm) was used as cathode. It was immersed in the electrolyte which was consisted of ethylene glycol (EG 5%) dissolved in sulfuric acid (H_2_SO_4_ 98%) and hydrofluoric acid (HF 40%).

The HF concentration was remained constant (1% (*v*/*v*)) to decrease the rate of anodization reaction and to strengthen the adhesion of the Ta_2_O_5_ NTs. 

The anodization experiments were conducted at 0 °C and 20 V with an anodization interval of 15 min. After the anodization, Ta_2_O_5_ NTs were rinsed using deionized water to exclude excessive H_2_SO_4_ and HF. To re-grow the Ta_2_O_5_ NTs, the anodization step was performed again under the same conditions for 5 min. As reported by previous study [39], the firstly synthesized nanostructured coating may exist some cracks and have the potential to delaminate, thus a two-step anodization technique was applied to develop a relatively dense coating and avoid severe cracks in this work. The surface of the anodized coating was then cleaned by sonication in ethanol and dried at 100 °C because of a color transformation from light gray to white. To enhance the initial adhesion, heat treatment was carried out at 450 °C for 1 h. This is attributed to promotion of inter diffusion between the nanotube and coating, and then stronger interlock of them.

### 2.3. Characterization of Coatings

The phase identification of the MNT was performed by an XRD instrument (D8 Discover, Bruker AXS Gmbh, Karlsruhe, Germany) over a 2θ range from 20 to 80° operating at 45 kV and 30 mA. The surface morphologies of MT and MNT coatings were characterized using a SEM (Hitachi S-4800, Tokyo, Japan). To characterize the cross-section of nanotube in the MNT coatings, a destructive approach through diamond cutting tool was employed. The close observations to the coatings were carried out on a TEM (JEOL JEM-2100F, Akishima-shi, Japan) operated at 160 kV. The roughness of these two coatings were characterized by a roughness measuring meter (MarSurf XCR 20, Göttingen, Germany). The contact angle of these two coatings was measured by using a Contact angle measurement (Zhongchen Company, JC2000-D, Shenzhen, China).

Electrochemical measurements of polarization curve were performed using a CS310 electrochemical workstation (Wuhan Corrtest Instruments Inc., Wuhan, China) in a standard three-electrode cell of 500 mL at 37 °C. MT coatings/MNT coatings were used as the working electrodes. The counter electrode was a quadrate platinum slice with surface area of 10 cm^2^. The reference electrode was a saturated calomel electrode (SCE). Polarization curves of MT coatings and MNT coatings were carried out in simulated body fluid (SBF) at a scan rate of 0.1 mV/s.

The values of zeta potential of the coatings were measured by Surpass (Anton Parr, Graz, Austria). The pH buffer had 25 mL of 0.001 mol/L KCl solution and 75 mL of NANO pure water. HCl or NaOH solutions were added as necessary to adjust the pH.

### 2.4. Protein Adsorption Assay

Bovine serum albumin (BSA) and fibronectin (Fn) were served in the protein adsorption assay as model proteins. Both BSA (Sigma, Tokyo, Japan, 10 μg/mL) and Fn (Sigma, Tokyo, Japan, 10 μg/mL) contained in 1 mL of DMEM (Gibco, Fisher Scientific SAS, Illkirch Cedex, France) were added onto each sample in a 48-well plate. Two replicates were used for both MT and MNT groups. All the specimens were transferred, after incubation for 0.5 and 4 h respectively at 37 °C. Adsorbed protein on the specimen was detached by 500 μL sodium dodecyl sulfate (SDS) solution after being shaken for 1 h. The amount of protein adsorbed on the micro-scale tantalum coating (MT) and micro/nano scale tantalum (MNT) coating with Ta_2_O_5_ nanotubes (MNT) surface were measured by bicinchoninic acid (BCA) protein assay, respectively.

### 2.5. Cell Morphology

hBMSCs used in the research was purchased from Sciencell research laboratories (Sciencell, Carlsbad, CA, USA). Cells were seeded onto each specimen of MT and MNT with density of 2 × 10^4^ cells. Specimens were incubated for 4 days, and then washed twice by using PBS, followed by fixing in glutaraldehyde overnight, and subsequently cleaned again. To prevent the morphology deformation, ethanol with different graded series were used for cell dehydrating and dried in 37 °C overnight. In the end, the morphologies of samples were obtained using SEM after gold coated.

### 2.6. Cell Cytoskeleton Immunofluorescence

By means of confocal laser scanning microscopy (LSM880NLO FILM, Carl Zeiss, Jena, Germany), the cell cytoskeleton on specimens was investigated. After incubating for 24 h, each specimen was cleaned twice by using PBS, and subsequently fixed with paraformaldehyde for 20 min. They were immersed in 1% BSA solution for 1 h after washing three times. Then, the cells were stained with rhodamine-phalloidin for 45 min. Cell nuclei were treated with 5 g/L of 40, 60-diamidino-2-phenylindole (DAPI,) for 10 min in dark after rinsing for three times. Finally, cells were studied under confocal microscopy after thorough washing with PBS.

### 2.7. hBMSCs Adhesion and Proliferation

Cells were seeded onto each sample of MT and MNT, and incubated for 1, 4 and 7 days. The studies of attachment and proliferation of hBMSCs were performed with the Cell Counting Kit-8 (CCK-8, Dojindo, Kumamoto, Japan). Then, 1 mL of cell suspension in DMEM added with 10% fetal bovine serum (FBS) and 1% antibiotics were seeded on MT and MNT. After each time point of cultivation times of 4, 8, 12 h, the solution was changed into 0.9 mL culture solution and 0.1 mL CCK-8.

### 2.8. Matrix Mineralization

There were 5 × 10^4^ cells seeded into each well in the 48-well plates. The cells were washed two times with PBS after culturing for 14 and 21 days. Later, the cells were fixed by adding 4% paraformaldehyde for 15 min at 300 K. After that, the cells were stained for 10 min at 300 K. The reagent is 0.1% alizarin red (Sigma) in Tris–HCl (pH 8.3). In the end, sodium phosphate containing 10% acetylpyridinum chloride of 10 mM was used for the dissolution of stains on the samples. Finally, a proper amount of solution was taken, and the respective absorbance was tested at a wavelength of 590 nm.

### 2.9. Osteocalcin Secretion

The expression of osteocalcin was tested by using an Elisa kit. Firstly, 5 × 10^4^ hMBSCs were seeded onto the samples of MT and MNT. Osteocalcin was collected and measured in a way that the protein concentration tested by Micro-BCA kit after the cultural time of 14 and 21 days.

### 2.10. Real-Time PCR

The osteogenesis-related gene expression was tested. Cells were seeded on the samples of MT and MNT. After incubating for 14 days, total RNA was isolated via trizol (Life Technology, Carlsbad, CA, USA). Quantitative real-time PCR was detected by an ABI 7500 Real-Time PCR System (Applied Biosystems, Foster City, CA, USA). The relative gene expression of osteogenic differentiation, namely Runt-related transcription factor 2 (Runx2), Osteocalcin (OC), and osteopontin (OPN) were analyzed. The β-actin was provided as a control for normalization in our work. Table 1 described the sequences of primers in forms of forward and reverse.

### 2.11. Statistical Analysis

The above experiments were conducted for 3 times. Errors estimated of all the data were presented by using standard deviation. By using methods of *t*-test, statistical differences were analyzed. It was regarded statistically significant that values of p should be less than 0.05.

## 3. Results and Discussion

### 3.1. Surface Characterization

**Figure 1:**
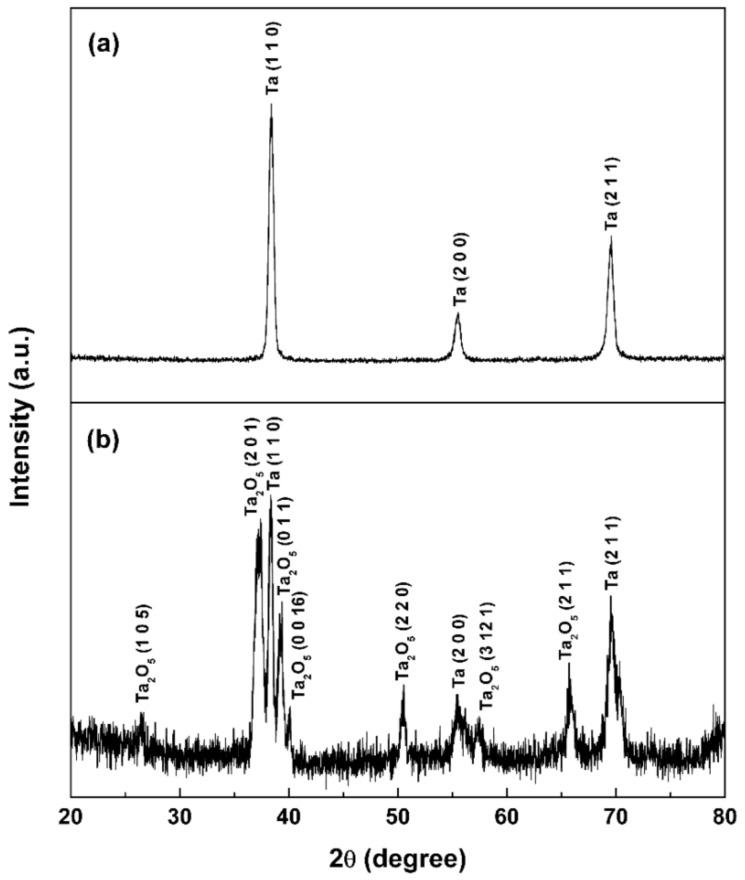
GIXRD profiles of the surface of as-sprayed tantalum (MT) coating (**a**) and surfaces obtained upon anodizing micro/nano tantalum (MNT) coating (**b**).

Figure 1 displays the grazing incident X-ray diffraction (GIXRD) profile of the MNT coating, and for comparison, the GIXRD pattern of the MT coating is also illustrated in Figure 1. It can be seen that three diffraction peaks appear at 2θ = 38.47°, 55.55° and 69.58° on the XRD profile of the MT coating, which corresponds to the planes of (110), (200) and (211) of Ta with a cubic structure (JCPDS#001-1182, Im-3m), respectively, implying that the MT coating mainly contains Ta phases (see Figure 1a). While for the MNT coating, the distinctive peaks mainly come from the tantalum oxide Ta_2_O_5_, indicating that oxidation of Ta occurred in the anodizing process (see Figure 1b).

**Figure 2:**
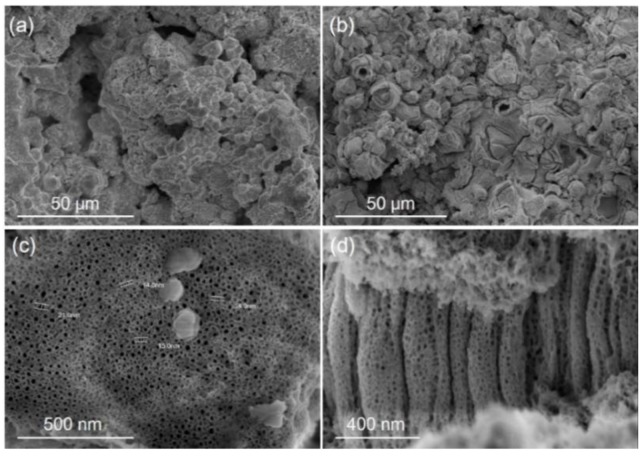
SEM images of as-sprayed tantalum (MT) coating surface (**a**); anodizing micro/nano tantalum (MNT) coating surface (**b**); cross-section by mechanical fracture of the sample (**c**); top view of resulting nanotube array (**d**).

Figure 2 illustrates the surface morphologies of the MT and MNT coatings. The MT and MNT coatings exhibit a rough surface with a porous structure (see Figure 2a,b). Close observations reveal that the surface of the MNT coating is covered with nanotube arrays, as seen in Figure 2c,d. From the top and cross section views, one can measure the dimensions of the nanotubes, which are about 15 nm in diameter and 800 nm in length. They can be formed by tuning the growth conditions such as anodization potential and anodization time, as revealed by other studies [36,40].

**Figure 3:**
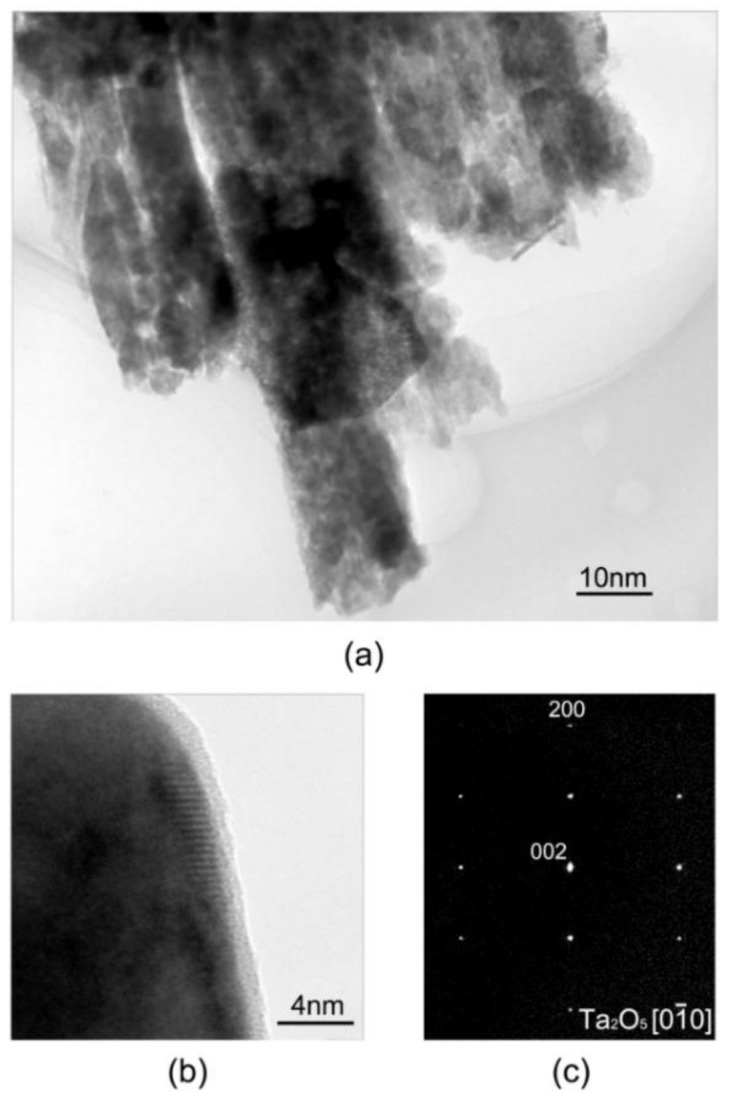
TEM image and high resolution TEM image of nanotube(s) (**a**,**b**); the SADPs taken from the orthorhombic Ta_2_O_5_ phase (**c**) (the zone axis indices are indicated).

Furthermore, closer observations to the nanotubes were conducted on a High-resolution transmission electron microscopy (HRTEM). Figure 3 shows the bright field TEM image of nanotubes, and the corresponding selected area diffraction pattern (SADP). Nanotube with diameter of 15 nm can be observed. The selected-area electron diffraction (SAED) pattern from agglomerated nanotubes showed [$0\bar{1}0$] diffractions of the orthorhombic phase of tantalum (See Figure 3). From it, we can deduce the phase has an orthorhombic structure with lattice parameters of a = 0.099 nm, b = 0.100 nm, and c = 0.362 nm. The results are consistent with that reported in the literature [41].

**Figure 4:**
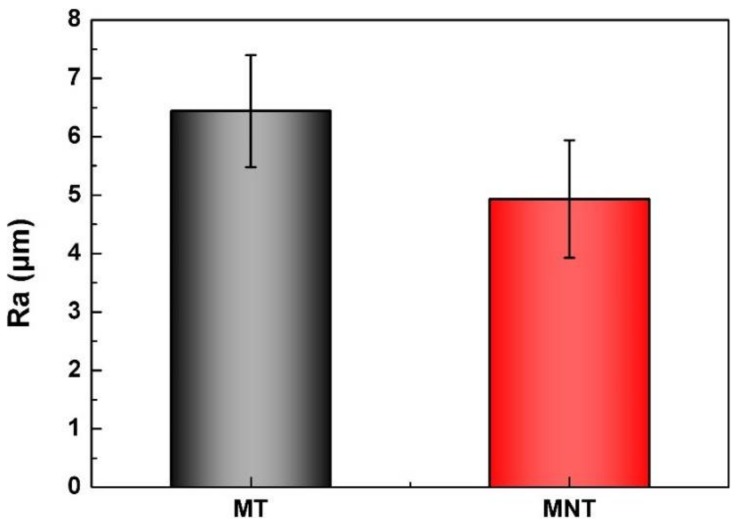
The roughness of MT and MNT coatings.

Changes in surface roughness usually result in changes in cell morphology, adhesion, and proliferation. The values of roughness of MNT and MT coatings have been measured. As it can be seen from Figure 4, the roughnesses of MNT and MT coatings are measured to be 6.44 ± 0.96 nm and 4.93 ± 1.00 nm, respectively. That is to say, the surface roughnesses have no significant change after anodic oxidation. The hydrophilicities of these two coatings are characterized with contact angle in the follows.

**Figure 5:**
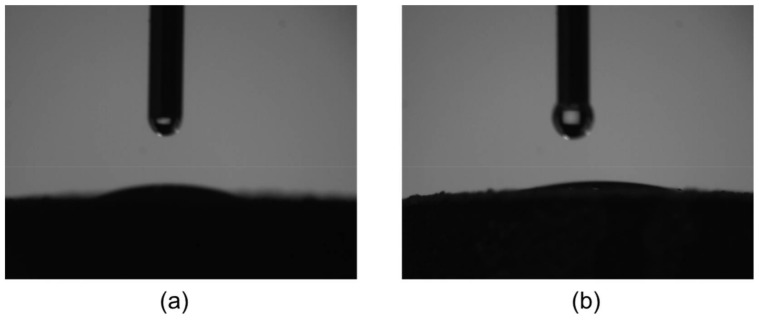
The contact angle measurement of MT (**a**) and MNT (**b**) coatings.

Figure 5 shows the H_2_O contact angles of MNT and MT coatings. The contact angles of MT and MNT coatings were 15.6° ± 0.5° and 12.1° ± 0.9°, respectively. MNT coating is more hydrophilic than MT coating with a slightly lower contact angle. Note that during the cell incubation, the interaction of the cell membranes with the surface of the coating may partly determine by the hydrophilicity, the lower contact angle with hydrophobic surfaces of MNT coatings could lead to better cell attachment.

### 3.2. Electrical Performance

**Figure 6:**
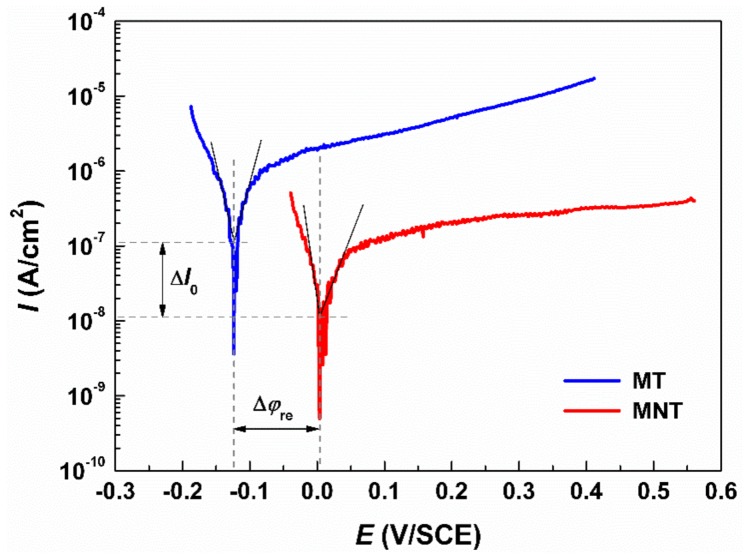
Polarization plots of as-sprayed (MT) coating and micro/nano tantalum (MNT) coating. The intersections of the anodic and cathodic Tafel slopes of the polarization curves indicate the corrosion potentials (*Ψ*_re_) and corrosion currents (*I*_o_). The test solution is SBF at room temperature.

As a biomaterial, it is expected to keep biologically innocuous during functional period. Hence, corrosion resistance related to chemical stability is a significant metric which can affect the biocompatibility of a metallic biomaterial. Figure 6 shows the polarization curves of MT coating and MNT coating. The results show that the corrosion potential and corrosion current density of MT coating are −0.124 V (vs. SCE) and 10^−7^ A/cm^2^, respectively. Moreover, the corrosion potential and the corrosion current density of MNT coating are measured to be −0.004 V (vs. SCE) and 10^−8^ A/cm^2^, respectively.

In contrast with MT coating, the MNT coating exhibits an improved corrosion resistance after anodization, which is evidenced by its polarization curve (see Figure 6). It is attributed to the formation of the Ta_2_O_5_ nanotubes on the surface of the tantalum coating. The dense coating over metallic substrate provides a barrier to prevent the metal ions releasing from matrix to electrolyte. Thus, in comparison with MT coating, of the MNT coating with Ta_2_O_5_ nanotubes decorated with micro/nano structure can promote corrosion resistance in SBF significantly. The enhanced chemical stability consequently give rise to an excellent biocompatibility of the samples. Therefore, it is reasonable to deduce that the Ta_2_O_5_ nanotubes decorated with micro/nano structure might avoid cytotoxicity, and chronic inflammation.

**Figure 7:**
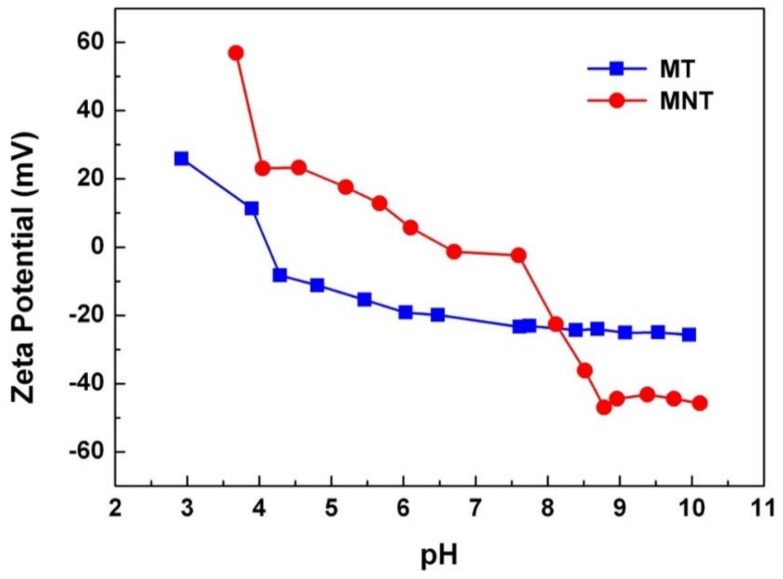
The Zeta potential of MNT and MT coatings.

The surface charge property of surface is another factor which can influence the adsorption of protein in the physiological environment [42]. The measurement results were showed in Figure 7. The isoelectric point of MNT and MT coatings are 6.58 and 4.12, respectively. Besides, the zeta potential of MNT and MT coatings at pH = 7.4 were −2.20 and −22.72 mV, respectively. The zeta potential of MNT coating was higher than that of MT coating in SBF. The lower amount of negative charges on the MNT coating would enhance the adsorption of protein which was negatively charged.

### 3.3. Protein Adsorption

**Figure 8:**
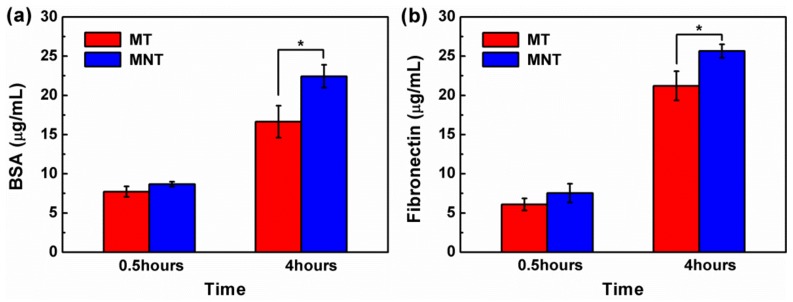
Protein adsorption on MT and MNT surface after incubation for 0.5, 4 h (bovine serum albumin (BSA) (**a**); and fibronectin (Fn) (**b**)). (* represents *p* < 0.05).

It is well documented that the cells response to a material is strongly dependent on the adsorption of the proteins from serum on the material surface [43]. Serum albumin is the major transport protein in blood plasma, which plays a vital role in regulating osteoblastic cell growth and function, such as promoting cell proliferation [44,45]. Moreover, integrin-fibronectin (Fn) interactions play a significant role in osteoblastic function, providing a structural framework for cell attachment, spreading, and differentiation through integrin receptors [46]. Therefore, the adsorption of BSA and Fn protein is studied, as shown in Figure 8. After 4 h, the surface of MNT displays a significant higher percentage adsorption of BSA and FN, which is mainly attributed to its nanoscale topography and hydrophilicity. It has been well confirmed that the nanostructures enhanced unfolding of the adsorbed proteins, promoting the interaction with specific receptor [47]. Meanwhile, the lower amount of negative charge of MNT can partly enhanced its adsorption of these two negatively charged proteins of BSA and Fn. Besides, the amount of adhesive protein Fn is greater than that of non-adhesive protein BSA for both MT and MNT coatings. During bone implantation, plasma fibronectin is deposited to form a blood clot between the implant and bone cavity, which help bone mesenchymal stem cells (BMSCs) migrate to the surface of the implant and differentiate [48]. Therefore, the higher adsorption capacity of Fn on the surface of MNT coating indicates that the MNT coating may enhance osteoinductivity and promote the osteogenesis of implants.

### 3.4. Cell Morphology and Cytoskeleton Fluorescence Staining

**Figure 9:**
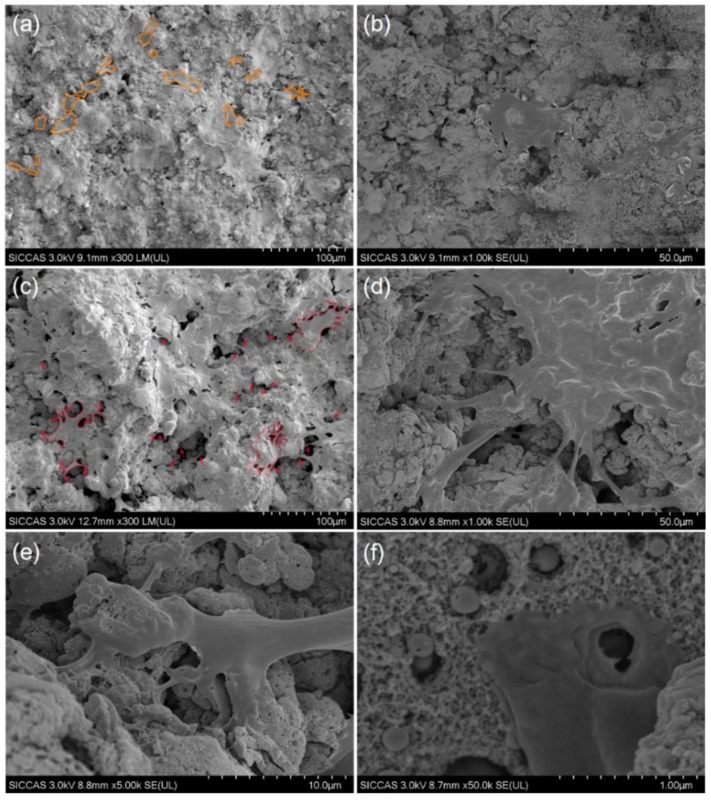
SEM images of morphology of cells after 4 days on (**a**,**b**) MNT coating and (**c**–**f**) MNT coating under different magnifications. From (**b**), cell on MT coating presents spindle morphology with less stretched morphology. From (**d**), it shows fully spread cell with well-stretched morphology and a relatively large size on MNT coating. From (**e**), it presents a good interaction between the nanotube surface and the filopodia of hBMSC; From (**f**), a filopodia presents a good interaction with the MNT surface. (The orange outlines indicate the approximate morphology of cells on the surface of MT coating. The red arrows mark the filopodia of hBMSCs on MNT coating.).

More nano-scale Fn is absorbed on the surface of MNT coating in comparison with MT coating, implying that the rough micro-/nano-scale structure of the surface of MNT coating may enhance the cell adhesion. Figure 9 show the SEM images of the morphology of cells after a culture of 4 days on the surfaces of the MT and MNT samples. The morphologies of hBMSC on the surfaces of MT and MNT coatings display the dramatically different shapes after 4 days’ incubation. On the surface of MT coating, a large number of cells present spindle or round shape with few filopodia (Figure 9a). In contrast with the cells on the MT coatings, the cells show higher degree of spreading and interacting with each other on the MNT surface with the introduction of nanotube, indicated by the polygonal morphology of the cells with a relatively larger size (Figure 9c). Close observation reveals that the filopodia is in abundance on the MNT surface (Figure 9d,e).

Furthermore, the cells show an elongated shape, acclimatized to the complex rough morphology, but poor cell attachment with few filopodia can be observed on the MT coating (Figure 9a,b). However, the cells spread well on the surface of MNT coating, as shown in Figure 9f. It can be seen that the MNT coating can provide the deformation and well-spread morphology of cells at the same time. When the nanotubes were introduced, the micro-porous topography could be maintained. Furthermore, the MNT coating can exert its influence in special ways. Nanotubes on the MNT coatings contribute to cell spreading, and the micro-porous surface of the coating provides the rough morphology, beneficial to unfolding of the cells in the meanwhile [34]. Hence, the synergistic effect is expected to obtain by the MNT coating.

**Figure 10:**
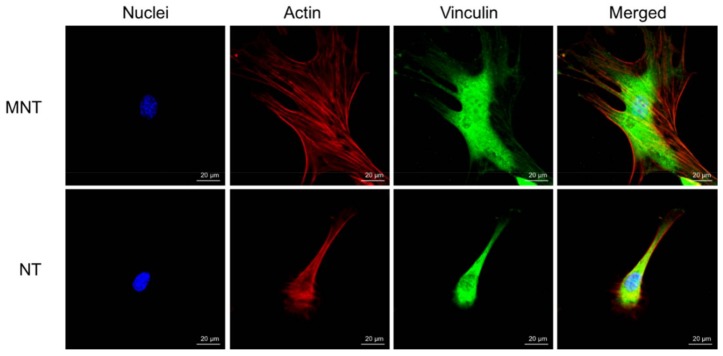
Fluorescence staining images of MC3T3 cells after 4 days on different coatings. Blue, nuclei; Red, actin; Green, Vinculin. “Merged” represents the merged pictures of the above three fluorescence.

The cytoskeleton fluorescence staining of two groups are shown in Figure 10. Extension of cytoskeleton can be observed on the MT coating while the edge of cells is relatively smooth edge. As for the MNT coating, the cytoskeletal stretching is well developed, and a large amount of filopodia can be observed.

As a result, the hierarchical micro/nano structure can enhance cell spreading, filopodia growth as well as multidimensional cytoskeleton distribution.

### 3.5. Cell Attachment and Proliferation

**Figure 11:**
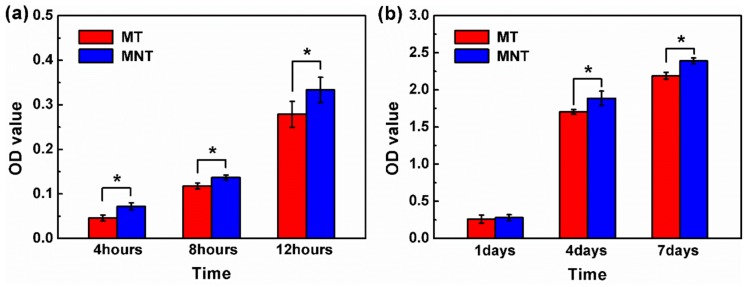
Adhesion of the hBMSCs on MT and MNT after 4, 8, 12 h (**a**); proliferation of hBMSCs on MT and MNT after 1, 4, 7 days (**b**). (* represents *p* < 0.05).

Cell attachment to the surface is essential for osteoblasts to spread, differentiate and finally form new bone. However, higher adhesion does not supportive to indicate that the cells are viable [35]. Therefore, the attachment and proliferation of hBMSCs cultured on the MT coating and MNT coating were evaluated by cck8 assay in the present work, and the results are illustrated in Figure 11. From it, one can see that a slightly larger number of cells are adhered on MNT than on the MT coating as the incubation time increases from 4 to 12 h, implying that hBMSCs are easy to be adhered on the surface of MNT coating due to plenty of protein adsorbed on it. The proliferation of cells on both surfaces of MT and MNT coatings exhibit a high proliferation rate, i.e., the culture time is from 1 day to 4 days, the cells on the surfaces of both the MT coating and MNT coating exhibit high proliferation rates, which reach 5.56 for MT coating and 7.58 for MNT coating, respectively. Therefore, the cells on the MNT coating exhibits a higher growth rate than that on the MT coating in the early 4 days. After culturing for 4 days, a higher density of cells is observed on the MNT coating than that on the MT coating, indicated by the cell morphology in Figure 9a,b. The growth rate of the cells on the MNT coating is approximately 36.33% more than that on the MT coating. The results indicate that the MNT coating exhibited more positive effects on hBMSCs proliferation compared with the MT coating. Studies have reported that there is a higher amount in integrin activation in the nano-topographic surface, and as a result in cell behavior of attachment and proliferation [49]. On the other hand, higher corrosion resistance of MNT coating which performs enhanced corrosion resistance might do help to prohibit cytotoxicity, and chronic inflammation may have an additional effect on the attachment and proliferation of hBMSCs [50]. As a result, the surface of the MNT coating exhibited much better cell-material interactions and improvement of the cytocompatibility which are necessary for differentiation and osteoblasts in a later stage.

### 3.6. Extracellular Matrix Mineralization and Osteocalcin Secretion

**Figure 12:**
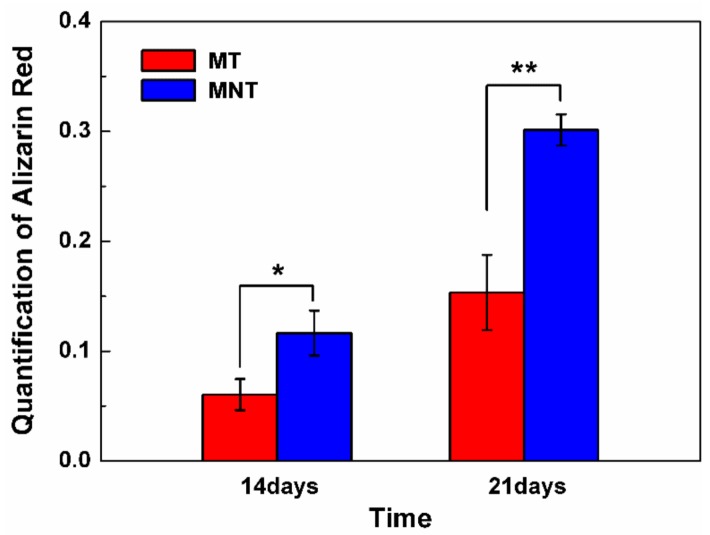
Comparative quantification of alizarin red on the surfaces of MT and MNT coatings after 14 and 21 days. (* represents *p* < 0.05, ** represents *p* < 0.01).

**Figure 13:**
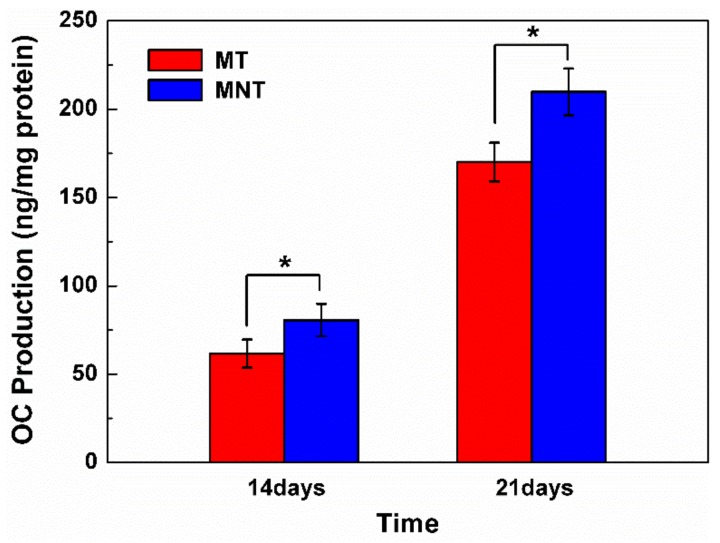
Comparative quantification of osteocalcin secretion on different surfaces of MT and MNT coatings after 14 and 21 days. (* represents *p* < 0.05).

As hBMSCs differentiate, they begin to deposit bone matrix which is dominant with calcium phosphate on the surface. Therefore, the amount of calcium deposition is measured by quantification of alizarin red staining on the surfaces of MT and MNT coating. As it can be seen from the result in Figure 12, the MNT coatings enhance mineralization compared with MT coating. The calcium content increases by approximately 100% on the surface of MNT coating for both 14 and 21 days of culture. Additionally, the osteocalcin secretion is assayed by ELISA and the results are shown in Figure 13. There is a similar tendency to the calcium deposition assay. The MNT coating with nanotubes provides a desirable interface for the cell differentiation and the matrix production. These results indicate that the combination of the micro pores and nanotube topography of MNT coatings can promote the osteogenesis.

### 3.7. Osteogenesis-Related Genes Expression

**Figure 14:**
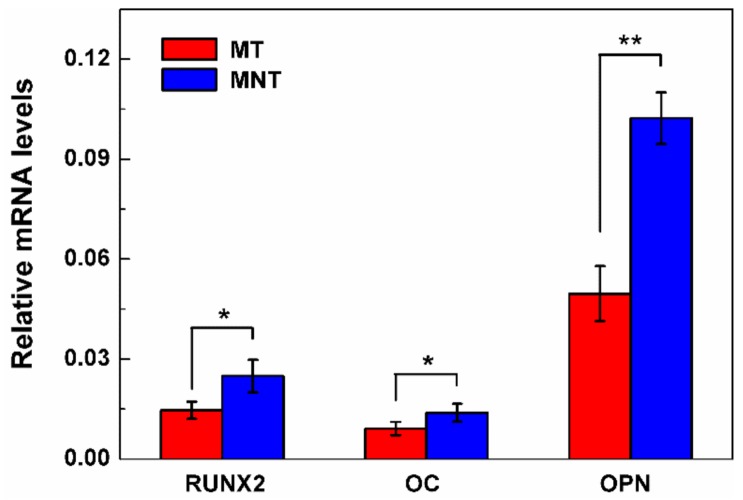
Relative expression of Runx2, OC and OPN in hBMSCs seeded on MT or MNT coated-surface, hBMSCs have a cultivation of 14 days in osteoinduction medium. All values normalized to beta-actin. (* represents *p* < 0.05, ** represents *p* < 0.01).

To further investigate the combined effects of micro-porous and nano structure of MNT coating on osteoblast differentiation at the molecular level, mRNA expression of Runx2, OC and OPN were characterized by PCR after 14 days. The results are illustrated in Figure 14. Runx2 has been identified as a key transcription factor regulating osteogenesis, and it is the controlling gene in early osteogenic differentiation stage [51,52]. OPN is an indicator for middle-stage differentiation, while OC is an indicator for late-stage differentiation. Both have positive effects on bone mineralization as noncollagenous matrix proteins [53,54,55,56].

As shown in Figure 14, Runx2, OC and OPN of osteoblasts cultured onto MNT coating are 1.71, 1.52, and 2.06 times higher than those of MT coating, respectively. It suggests that the surface with the Ta_2_O_5_ nanotubes can enhance hBMSCs differentiation and matrix formation on tantalum coating surface. The surfaces of the MNT coating can encourage the high expression level of OPN in our work, meaning that Ta_2_O_5_ nanotubes can improve hBMSCs matrix protein formation and osteogenesis regulation. The same trend for Runx2 is also observed. As a result, the MNT coating with the combination of micro-porous and Ta_2_O_5_ nanotubes can improve the differentiation of hBMSCs into osteoblasts, which can accelerate the osteogenesis response on the tantalum coating.

## 4. Conclusions

In this work, the micro/nano structural tantalum (MNT) coating was successfully prepared by using anodic oxidation combined with atmosphere plasma spraying method. The surface morphologies, corrosion, and biological performances have been comprehensively studied. The porous tantalum (MT) coating is chosen as a control group. The experimental results are listed as follows:

(1) The Ta_2_O_5_ NTs with diameter of about 15 nm are deposited on the micro-porous plasma-sprayed tantalum coating by a two-step anodization technique.
(2) The corrosion resistance of MNT coating has been enhanced by approximately one order of magnitude, because the tantalum oxides covered on the surface can work as a barrier to decrease the release of metal ions to the solution of the SBF.
(3) The MNT coating exhibits better cell spreading of hBMSCs and improved cytocompatibility in vitro. Moreover, it can enhance the hBMSCs differentiation which provides 1.5~2.1 times improvement in gene expression compared with the MT coating.

It suggests that the hierarchical micro/nano structure of tantalum coating exhibit a very promising application prospect in improving the osteointegration of the implant.

## Figures and Tables

**Table 1:** The sequences of forward (F) and reverse (R) primers for real-time polymerase chain reaction (PCR).

Target Gene	Direction	5′-3′ Primer Sequence
β-actin	F	5′-CATGTACGTTGCTATCCAGGC-3′
	R	5′-CTCCTTAATGTCACGCACGAT-3′
Runx2	F	5′-TGGTTACTGTCATGGCGGGTA-3′
	R	5′-TCTCAGATCGTTGAACCTTGCTA-3′
OC	F	5′-CACTCCTCGCCCTATTGGC-3′
	R	5′-CCCTCCTGCTTGGACACAAAG-3′
OPN	F	5′-CTCCATTGACTCGAACGACTC-3′
	R	5′-CAGGTCTGCGAAACTTCTTAGAT-3′
